# Nutritional Composition and In Vitro Starch Digestibility of Crackers Supplemented with Faba Bean Whole Flour, Starch Concentrate, Protein Concentrate and Protein Isolate

**DOI:** 10.3390/foods11050645

**Published:** 2022-02-23

**Authors:** Manu Pratap Gangola, Bharathi Raja Ramadoss, Sarita Jaiswal, Hrvoje Fabek, Mehmet Tulbek, Gerald Harvey Anderson, Ravindra N. Chibbar

**Affiliations:** 1 Department of Plant Sciences, College of Agriculture and Bioresources, 51 Campus Drive, University of Saskatchewan, Saskatoon, SK S7N 5A8, Canada; gangola.manu@gmail.com (M.P.G.); bharathiraja_6@yahoo.co.in (B.R.R.); sarita782000@gmail.com (S.J.); 2 Department of Nutritional Sciences, Faculty of Medicine, University of Toronto, 5th Floor, Medical Sciences Building, 1 King’s College Circle, Toronto, ON M5S 1A8, Canada; hrvoje.fabek@utoronto.ca (H.F.); harvey.anderson@utoronto.ca (G.H.A.); 3 Saskatchewan Food Industry Development Centre, Saskatoon, SK S7M 5V1, Canada; mtulbek@foodcentre.sk.ca

**Keywords:** cracker, faba-bean, resistant starch, starch hydrolysis, digestibility

## Abstract

The nutritional quality of common wheat-based foods can be improved by adding flours from whole pulses or their carbohydrate and protein constituents. Faba bean (*Vicia faba* L.) is a pulse with high protein concentration. In this study, prepared faba bean (FB) flours were added to wheat based baked crackers. Wheat cracker recipes were modified by substituting forty percent wheat flour with flours from whole faba bean, starch enriched flour (starch 60%), protein concentrate (protein 60%) or protein isolate (protein 90%). Baked crackers were ground into meal and analyzed for their macronutrient composition, starch characteristics and in vitro starch hydrolysis. Faba bean supplemented crackers had lower (*p* ≤ 0.001) total starch concentrations, but proportionally higher protein (16.8–43%), dietary fiber (6.7–12.1%), fat (4.8–7.1%) and resistant starch (3.2–6%) (*p* ≤ 0.001) than wheat crackers (protein: 16.2%, dietary fiber: 6.3%, fat: 4.2, resistant starch: 1.2%). The increased amylose, amylopectin B1- chain and fat concentration from faba bean flour and starch flour supplementation in cracker recipe contributed to increased resistant starch. Flours from whole faba bean, starch or protein fractions improved the nutritional properties and functional value of the wheat-based crackers. The analytical analysis describing protein, starch composition and structure and in vitro enzymatic hydrolysis advance understanding of factors that account for the in vivo benefits of faba bean flours added to crackers in human physiological functions as also previously shown for pasta. The findings can be used to guide development of improve nutritional quality of similar wheat-based food products.

## 1. Introduction

Snacks are small portions of food usually consumed to satisfy cravings between meals [1]. Crackers are a form of crisp bread generally prepared from wheat flour and fat, and a popular snack. The popularity of crackers is not restricted to a specific age group, gender or nationality. The global cracker market in 2020 was valued at United States dollars (US$) 20.6 billion, which is expected to reach USD 28.60 billion in 2027, an average growth rate of 4.8% (Verified Market Research^®^, 2021). The market demand for snacks is steadily increasing due to busy lifestyles and hectic schedules that necessitate consumers supplant traditional meals with healthy snack options. A recent survey published by Mondelez International showed consumers prefer snack foods that are high in protein and dietary fiber, but low in fat, salt, cholesterol, sugar and calories [2] The demand for healthy snacks presents an opportunity to increase the nutritional value of crackers [2,3,4,5]. Pulses are dry edible seeds of the Leguminosae family, excluding those used for oil extraction [6], cultivated globally over 96 million hectares producing on average more than 92 million tons per year [7]. The common pulses include lentil, chickpea, dry peas and beans which are starchy seeds that provide a wide array of nutrients including complex carbohydrates and high protein content that ranges between 17–35% [8]. Pulse protein is also high in lysine, an essential amino acid, that is low in cereal proteins [9]. Pulse carbohydrates contribute one-half to two-thirds of the seed weight and are composed of slowly digestible carbohydrates and dietary fiber, which results in low post-prandial glycemia compared to other carbohydrate rich foods such as white bread, rice or potatoes [10,11]. Pulse carbohydrate and protein characteristics combined with their minerals, vitamins and bioactive compounds concentrations makes them an attractive grain to supplement wheat-based products to enhance their nutritional quality [12,13,14,15].

Pulses are nutrient dense but have relatively low energy density (1.3 kcal/g, based on a cooked serving) [16], contributing to their health benefits [17]. Pulse rich diets have been associated with reduced incidence of chronic diseases including obesity, type 2 diabetes, cardio-vascular diseases, inflammatory diseases, and cancer [18,19,20,21,22]. Pulses are consumed in different forms and levels across countries, ethnic groups and economic classes [23]. In Asian countries, pulses are traditionally consumed as whole grains or after splitting (dahl), but in the Western countries, pulse flours are used to partially substitute wheat flour in pasta, bread and bakery products [15,23,24,25,26]. However, utilization of pulses to enhance the nutritional value of wheat-based products presents specific challenges for consumer acceptance that is dependent on texture, flavor, appearance, and color [5]. Elimination or reduction in antinutritional factors such as trypsin inhibitors, raffinose family oligosaccharides (RFO), phytic acid and tannins is required [5].

Among the pulses, faba bean (*Vicia faba* L.) has higher protein concentration than most, thus it is a preferred source of ingredients to enrich wheat-based products such as pasta and crackers. Faba bean protein concentration (28–32%) is higher than field peas (24%), low in oil, and a rich source of minerals, vitamins, and other micronutrients [8,27]. To improve pasta protein concentration, faba bean flour, starch concentrate, protein concentrate or isolate have been used for partial (10 to 50%) replacement of durum wheat semolina [15,21,28,29]. Compared to the native faba bean flour, fermented faba bean flour markedly improved the protein quality and bioavailability [30] as well improving baking quality in bread while moderately increasing its starch digestibility [31].

The inclusion of pulses in human diets contribute to dietary management of obesity and diabetes [18,28,30,32,33]. Canada’s food guide (2019) [34] and planetary health diet [35] emphasize inclusion of plant-based protein in diets. Canada is also one of the largest producers of pulses and the percentage of people consuming pulses has increased from 6.2% to 13%, but it has not become part of mainstream diets [36]. Strategies to increase pulse consumption include introducing cereal and pulse combinations in meal options, and snack foods [37].

The effect of processing pulse flours with wheat flour to create new wheat-based products on composition, carbohydrate structure and starch digestibility has had limited investigation [15,38]. We described in a previous report the effect of faba bean fortification of durum wheat pasta on protein, starch composition and structure and in vitro starch digestibility [15]. In this study, four different faba bean flours derived from the same variety but different fraction grade (flour/starch/protein concentrate/protein isolate) were tested in a cracker formula and compared with wheat cracker. An in vivo study has shown that the faba bean supplemented crackers had lower post-prandial glycemia [39]. The purpose of this report is to provide an analytical understanding of the characteristics of protein content, starch composition and structure and in vitro enzymatic hydrolysis that may account for the in vivo benefits of faba bean flours added to crackers [39] on post-prandial glycemia and human physiological functions as was also previously shown for pasta [15]. The findings can be used to guide development of improved nutritional quality of similar wheat-based food products.

## 2. Materials and Methods

### 2.1. Flour Formulation for Crackers Preparation

Faba bean (FB) flour, starch concentrate, protein concentrate and protein isolate flours were supplied by AGT Food and Ingredients (Saskatoon, SK, Canada) (Table 1). FB flour was milled from the whole dehulled and split faba bean grains [cv Malik (FB9-4)] using standard processes of drying, grinding followed by water and air fractionation techniques. Four types of PulsePlus™ faba bean flours were used to replace 40% of wheat flour in cracker recipes. PulsePlus™ faba bean V-6000™ is starch enriched faba bean flour, PulsePlus™ faba Bean Protein 60 contained ~60% protein while Protein 90 a protein concentrate contained ~90% protein (Table 1).

### 2.2. Methods

#### 2.2.1. Protocol for Cracker Manufacturing

**Figure 1:**
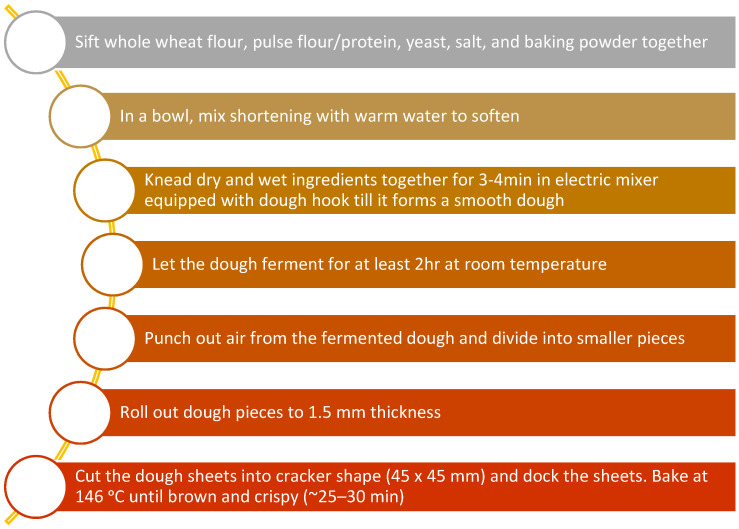
General flow chart of cracker preparation.

**Figure 2:**
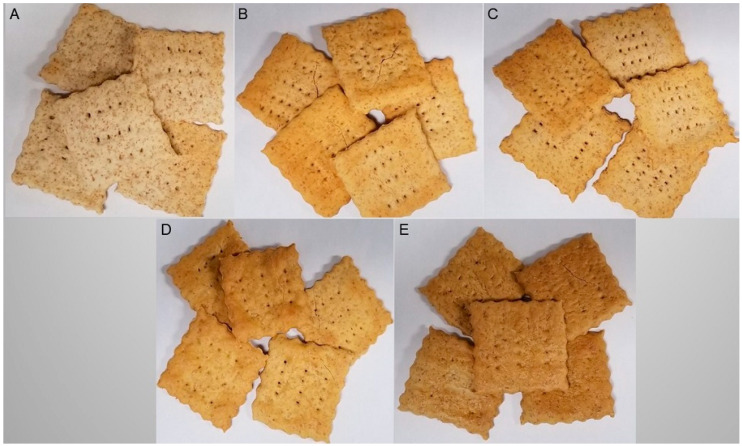
Crackers prepared using only wheat flour (**A**), 60% wheat flour + 40% PulsePlus™ faba bean flour (**B**), 60% wheat flour + 40% PulsePlus™ faba bean Flour V-6000™ (**C**), 60% wheat flour + 40% PulsePlus™ faba bean protein 60 (**D**) and 60% wheat flour + 40% PulsePlus™ faba bean protein 90 (**E**).

Cracker dough was prepared by combining 90 g wheat (*Triticum aestivum* L.) flour with an average of 2.8 g instant yeast, 1.2 g salt, 1 g baking powder, 5 g shortening and 45 g water to achieve a uniform taste and texture (Figure 1 and Table 2). In the FB supplemented crackers, 40% of wheat flour was replaced with respective FB flour or concentrates (Table 2). After kneading, rolling and cutting the raw control wheat crackers and faba bean crackers were baked in a deck oven (Doyon Inc., Menominee, MI, USA) at 146 °C for 15 min (Figure 2). An internal trained and experienced panel at the AGT Foods and Ingredients (Saskatoon, SK, Canada) found the texture to be similar in the control (CC) and FB supplemented crackers.

#### 2.2.2. Preparation of Cracker Meal for Proximate Analysis

For proximate analysis, wheat (control) and FB- (flour/starch/protein concentrate/protein isolate) supplemented cracker samples were ground using pestle-mortar and passed through a 0.5 mm sieve using Udy Cyclone Mill (Udy Corporation, Fort Collins, CO, USA) to produce ground meal samples, stored in a desiccator till used for analysis.

#### 2.2.3. Proximate Analysis in Cracker Meal Samples

Ground meal samples obtained from control wheat and FB- (flour/starch/protein concentrate/protein isolate) supplemented crackers were used to determine the total starch concentration [40], protein [41], crude fat concentration [42] and total dietary fiber (TDF) [43] as previously described. [15]. Amylose concentration in these samples was determined by a concanavalin A method using a commercial kit (Megazyme Amylose/Amylopectin Assay Kit, Megazyme, Wicklow, Ireland) following the manufacturer’s instructions.

Total raffinose family oligosaccharides (RFO) concentration (mmol/100 g on fresh weight basis) in all samples was determined by stepwise enzymatic hydrolysis using a commercial assay kit (K-RAFGL 04/18, Megazyme International Ireland Ltd., Wicklow, Ireland) [44]. For quantitative RFO determination from, an alcohol-based extraction followed by purification, with C18 column was used. A CarboPac PA100 column attached to HPAEC-PAD (High Performance Anion Exchange Chromatography with Pulsed Amperometric Detector; Dionex Canada Ltd., Oakville, ON, Canada) was used to separate the soluble sugars and determine the respective RFO concentration as described [45].

#### 2.2.4. Cracker Starch Isolation, Purification, and Amylopectin Branch Length Determination

Ground wheat and FB supplemented (flour/starch/protein concentrate/protein isolate) cracker ground meal (0.2 g) was mixed in 5 mL deionized water to hydrate the sample matrix. After vigorous mixing the sample slurry was filtered through a nylon membrane (100 µm pore size). Filtered meal samples were centrifuged at 3000× *g* [Allegra^TM^ 6, Beckman Coulter, Indianapolis, IN, USA] for 10 min and supernatant was discarded. Protein and crude fat deposited on top of crude starch was scraped off using a spatula. The crude starch pellet was purified using 80% (*w*/*v*) cesium chloride density centrifugation followed by water, sodium dodecyl sulphate wash buffer and acetone washes as described earlier [46].

Fluorophore-assisted capillary electrophoresis (FACE) was used for amylopectin chain-length distribution analysis [47]. The labelled (using 8-aminopyrene 1,2,6-trisulfonate and 1 M sodium cyano borohydride/tetrahydrofuran) de-branched starch samples were incubated in the dark for 16 h. An aliquot (5 μL) of the diluted (40×) sample was used for analysis. Laser-induced fluorescence detector module with an argon laser 238 was the excitation source. Samples were separated (Proteome Lab PA800; Beckman Coulter, Fullerton, CA, USA) at constant voltage and temperature (30 kV, 10 °C) for an hour using NCHO (PVA) capillary with pre-burned window (50 μm ID and 50.2 cm total length).

#### 2.2.5. In Vitro Enzymatic Hydrolysis of Crackers

The analysis was performed in two phases to calculate digestible and non-digestible starch fraction in the sample. In the first phase, ground wheat and FB supplemented (flour/starch/protein concentrate/protein isolate) cracker samples (100 mg, in triplicates) were suspended in 4 mL of amylosis enzyme mixture (pancreatic α-amylase 10 mg/mL plus amyloglucosidase 3 U/mL in sodium maleate buffer 0.1 M, pH 6.0). The samples were incubated for 0, 20, 30, 60, 120, 240 min in separate tubes. Reaction was terminated using ethanol (4 mL, 99%, *v*/*v*) followed by centrifugation (3000× *g*, 15 min). Supernatant (digestible fraction) was collected in separate tube. In the second phase the residue pellet (non-digested fraction) was washed twice with ethanol (50%, *v*/*v*). After final ethanol wash and drying the non-digestible starch fraction was dispersed in cold potassium hydroxide (2 mL, 2 M) for 20 min. Further digestion of this alkaline suspension continued using amyloglucosidase (3300 U/mL) in 8 mL of sodium acetate buffer (1.2 M, pH 3.8). Both phases end products were analyzed using Megazyme Resistant Starch kit method (Megazyme, Wicklow, Ireland) following manufacturer’s instructions. Rate of starch digested (hydrolyzed) was expressed as the percent total starch (TS) at the end of each interval. The digested starch values obtained at 20 min, 120 min, and more than 120 min of incubation were used as rapidly digestible starch (RDS), slowly digestible starch (SDS) and resistant starch (RS) [48]. The kinetic constant (*k*) for the cracker meal digestion was calculated using the following first order equation:

$$C=C_{\infty }\;\left(1-e^{-kt}\right)$$

*C* refers to the percent starch hydrolyzed at time *t*, *C*_∞_ is the equilibrium concentration of starch hydrolyzed after 240 min, *t* is the selected time point and *k* is the kinetic constant [49]. The area under curve (*AUC*) for the hydrolysis was calculated using the equation:

$$(AUC=C_{\infty }\;\left(t_{f}-t_{0}\right)-\left(C^{\infty }/k\right)\;\left[1-exp\;\left\{-k\;\left(t_{f}-t_{0}\right)\right\}\right]$$

Here, $t_{f}$ final time point (240 min), *t*_0_ is the initial time point (0 min) and *k* is the kinetic constant.

HI was calculated by dividing the area under the hydrolysis curve of individual sample by the corresponding area of the control cracker sample.

### 2.3. Statistical Analysis

To analyze the difference in nutritional constituents among flour and cracker samples, pairwise comparisons were performed using Tukey’s method (*p* < 0.001) in Minitab 18.0 software (Minitab Inc., State College, PA, USA). These comparisons were performed separately for each category, i.e., flour and cracker samples. All other calculations were performed using Microsoft Excel version 365 ProPlus (Microsoft Canada Inc., Mississauga, ON, USA).

## 3. Results and Discussion

### 3.1. Proximate Analysis

Starch and proteins were the major constituents in crackers. Starch contributed 33% to 62%, while proteins accounted for 16% to 43% to the total weight (Table 2). All faba bean flour supplemented cracker samples contained significantly reduced (*p* < 0.001) starch concentrations compared to the control cracker sample (61.8%), with the exception of the FB-starch supplemented cracker (61.0%) with PulsePlus™ faba bean Flour V-6000™ (Table 2). Protein concentrations in faba bean crackers reflected the protein content of the added flours [15], and thus had higher protein concentration than the control crackers (Table 2). Most faba bean supplemented crackers had higher (*p* < 0.001) protein concentration (22–43%) than the control cracker (16%) except for the FB-starch (aba bean flour V-6000™) supplemented cracker. The higher protein concentration and quality in the other FB-supplemented crackers showed that pulses can be a value-added ingredient to formulate functional wheat-based foods. This is promising for the snack industry, which is looking for easily accessible healthy ingredient options. Faba bean flour has twice the protein concentration of wheat flour [50] and is higher in the essential amino acid lysine, which is the limiting amino acid in cereal grains. In contrast, pulse proteins are limiting in the sulphur containing amino acids, methionine, cysteine and cystine [51]. Therefore, a combination of wheat and pulse flours elevate the quantity of all amino acids thus enhancing the overall protein quality [52]. Extracted pulse proteins have good water absorption, emulsion stability and foaming characteristics that help pulse flours contribute to desirable texture attributes while enhancing the nutritional quality of wheat-based food products such as pasta, crackers and cookies [53]. The FB-flour and protein supplemented crackers appeared darker in color than wheat or FB-starch crackers (Figure 2). Tannins, which contribute to majority of total phenolic in faba bean may be responsible for the observed color differences [54].

Addition of the faba bean flours increased (*p* < 0.001) fat concentration of the crackers, with highest increase in FB-flour supplemented cracker, followed by FB-starch cracker and FB-protein concentrate cracker and the least in FB-protein isolate supplemented cracker. (Table 3). Fat concentration of crackers reflected the fat concentration in the flour fractions. The fat concentration in faba beans at 4% is double the in wheat at 2%.

Flour type used in baking process determine majority of the nutritional profile of baked product. FB-flour supplemented crackers showed highest fat content than other FB-starch/protein supplementing flour crackers. The decrease in carbohydrate fraction in case the FB-protein isolate and concentrate supplemented cracker accounted for their lower total fat content compared to FB-starch crackers (Table 1 and Table 2).

Faba bean seeds are rich in dietary fiber. As expected, faba bean supplemented crackers, especially FB-protein concentrate supplemented cracker showed significantly (*p* < 0.001) higher TDF concentration compared to the wheat only cracker sample (Table 3). Higher dietary fiber has been reported in cookies made with pulse flour including faba bean whole flour [38]. Higher dietary fiber in the FB-protein concentrate than in FB- protein isolate supplemented cracker was due to higher dietary fiber in the PulsePlus™ faba bean protein 60. These flours could help fill the fiber gap in Canadian diets through food innovation strategies that include foods and ingredients with added dietary fiber [55]. Fiber affects transit time and fecal bulk, which is an important aspect of gut health [4,56].

Crackers supplemented with faba bean remained unaltered in crunchiness and hard texture (data not shown). Thus, the addition of faba bean flour and protein fraction provide a promising option to increase the nutritive values of crackers [28,30,57] without altering their desired texture characteristics. With less time and resources required in protein concentrate processing than protein isolate, it would be the preferred flour to enrich fiber content of faba bean containing crackers.

Total RFO concentration ranged from 1.6 to 4.5 mmol/100 g in faba-bean flour samples [Table 4]. However, they were only present in trace amounts in faba bean cracker samples and hence not reported. Stachyose (4.2 and 5.5%) was the major RFO in PulsePlus™ faba bean flour and PulsePlus™ faba bean protein 60 followed by verbascose (3.8 and 3.8%) and raffinose (1.4 and 1.9%). However, in PulsePlus™ faba bean flour V-6000™ and PulsePlus™ faba bean protein 90, verbascose (3.0 and 2.3%) was the predominant RFO followed by stachyose (2.4 and 1.4%) and raffinose (0.8 and 0.2%), respectively (Table 4). RFO-related flatulence is a major consideration in the consumer hesitancy to accept pulse-based food products, [13]. However, cooking [58,59] and, in the case of crackers, leavening and baking, significantly reduced RFO concentrations to the limit of becoming undetectable in the HPLC-based highly sensitive quantitation procedure used in this study.

### 3.2. Cracker Starch Amylose and Amylopectin

Amylose content in the crackers reflected its content in the flours. Amylose concentration in FB-starch supplemented crackers was significantly (*p* < 0.01) higher (44.5%) than in the FB-protein concentrated crackers (36.9%) but similar to all other crackers (Table 3). Faba bean starch is higher in amylose than cereal starches [60]. Thus, substituting 40% of wheat flour with faba bean starch (PulsePlus™ faba bean Flour V-6000™) in crackers increased amylose concentration (Table 3).

The addition of faba bean starch altered the amylopectin glucan chain distribution. Crackers supplemented with faba bean whole or starch flours had lower concentration of A- but higher concentration of B- chains (degree of polymerization [DP] 6–12 and DP13–24, respectively) compared with the wheat crackers, but the protein concentrate or isolate additions to crackers did not significantly affect their concentration (Table 5). B2- and C- chains were also increased after addition of the FB-starch flour. This was expected as amylopectin is a component of wheat starch. Thus, starch structure reflected more similarity with cereal starch amylopectin compared to faba bean along with DP maxima (DP 11) typical for cereal starch [61]. The changes in amylopectin glucan chains in faba bean supplemented crackers follow the general trend observed in faba bean supplemented pasta [15], with one significant difference. The difference in amylopectin glucan chain pattern was highest with the addition of starch concentrate in FB-starch supplemented cracker while in pasta, the greatest difference was observed in pasta with faba bean flour [15]. These results could be due to either the different process used to make pasta and crackers or the different concentrations of faba bean flour supplementation in pasta (25%) compared to 40% in crackers. Clearly, both factors require consideration in product development.

### 3.3. In Vitro Starch Digestibility of Faba-Bean Cracker

Enrichment of wheat flour with FB-flour and FB-starch faba reduced the hydrolytic index (HI) of wheat crackers (Table 6). The kinetic constant (*k*) in supplemented crackers were not different from the wheat crackers but both *k* and HI were higher after enrichment with FB protein concentrate or isolate than after FB flour or starch additions (Table 6). In wheat crackers, RDS was the most prominent fraction, followed by SDS and a very small amount of RS. The addition of all FB flours to crackers reduced (*p* < 0.001) RDS compared to wheat crackers (61.4%) (Table 6). The reduction in RDS was accompanied by an increase in RS and increase in B1- and C-, and a decrease in A- type amylopectin chains (Table 5 and Table 6) indicates that starch amylopectin structure inside the product matrix influences overall product digestibility. The presence of long glucan chains has been associated with increased RS and reduction readily available starch in pulses [62].

### 3.4. Correlation between Cracker Meal Components and Digestibility Kinetics

Correlation analysis between starch digestibility parameters (HI, *k*, RDS, SDS and RS) and cracker constituents (total starch, protein, fat, amylose, TDF, amylopectin chains (A, B1, B2 and C) revealed that amylopectin chain B1- followed by A- chains had dominating influence on in vitro starch digestibility parameters (Table 7). Amylopectin A- chain concentration positively correlated with digestibility parameters HI (r, 0.796; *p* < 0.001), *k* (r, 0.626; *p* < 0.01) and RDS (r, 0.846; *p* < 0.001) while negatively with RS (r, −0.79; *p* < 0.001). Amylopectin B1- chains showed an opposite relation with digestibility parameters (HI, *k*, RDS and RS). Longer amylopectin chains provide more resistance during enzymatic hydrolysis which influences overall digestibility of the food matrix [62].

Other than amylopectin chains, fat concentration was the major cracker constituent influencing in vitro starch digestibility. Fat concentration positively correlated with RS (r, 0.628; *p* < 0.01) and SDS (r, 0.682; *p* < 0.01) indicating its role in reducing digestibility. Intra-correlation analysis within digestibility parameters showed significant correlation of HI with RS (r, −0.832; *p* < 0.001) while with SDS it was not significant. Hydrolysis kinetic rate constant *k* in contrast to HI showed significant correlation with SDS (r, −0.776; *p* < 0.001). RDS showed significant correlation with HI (r, 0.903; *p* < 0.001) as well as *k* (r, 0.675; *p* < 0.01). Amylose and fat showed negative relation with HI (Table 7). As amylose, concentration is higher in FB-flour/starch supplemented crackers, high RS observed in these cracker samples is probably associated with amylose concentration. Total dietary fiber (TDF) showed significant correlation only with *k* (r, 0.516; *p* < 0.01) and *k* in this analysis correlated with SDS only, which indicate TDF contributed to SDS in cracker samples.

These results concur with our previous study on faba bean supplemented pasta where we found that medium B1- chains [degree of polymerization (DP), 13–24] showed positive correlation with RS in raw starch samples but showed no correlation in the cooked pasta [15]. Pasta is consumed after cooking while ready to eat crackers require no further processing. This also supports the observed effect of faba bean starch fraction on the overall in vitro digestibility. However, in a human trial study [39] on similar faba bean flour supplemented crackers, protein concentrate and isolate were the primary components reducing post prandial blood glucose. Another major factor, which differentiate in vitro and in vivo study results, is the overall enzymatic reactions catalyzing the meal digestion.

Crackers are ready to eat. As a result, the benefits provided by faba bean flour supplementation are practical as there is no secondary influence of consumer cooking time or secondary processing. However, the health benefits that may be achieved will depend on the composition of the faba bean flour. Faba bean protein supplemented crackers (FB-protein concentrate supplemented cracker and FB-protein isolate supplemented cracker) with similar starch digestibility profile as the wheat cracker control also offer significantly higher protein concentration and quality and TDF to the consumer. Whereas faba bean flour and starch concentrate containing crackers provide a reduced digestibility profile. Both formulations can be used for post-prandial glucose management and weight management. Increased dietary fiber in FB-supplemented crackers can be useful to target fiber gap in Canadian population [55]. These characteristics of faba bean flour, starch, protein concentrate or isolate can further refine their use in functional food design targeted to specific end-users [30,63,64,65].

## 4. Conclusions

Faba bean whole, starch concentrate, protein isolate or concentrate flours use at 40% supplementation in wheat cracker formulations have the potential to add health benefits to consumer snacks. They differentially altered starch composition and amylopectin structure, in vitro enzymatic hydrolysis of wheat crackers. Changes in starch composition (amylose %) and structure (amylopectin chain-length distribution) combined with higher dietary fiber and fat decreased the overall starch digestibility of the crackers. The amylopectin B1- chain and fat concentration in faba bean supplemented crackers contributed towards RS, while amylopectin A- chain proportion in absence of faba bean carbohydrate fraction in crackers resulted in an increase in HI and RDS and, hence, overall digestibility. Amylose in FB-supplemented crackers contributed towards RS while dietary fiber influenced SDS. The protein concentrate, and isolate flour increased both protein quantity and quality of the wheat crackers. Therefore, faba bean supplemented crackers can be formulated using specific constituents such as protein and/or starch to improve their nutritional quality and human physiological functions to meet the end-user specifications.

## Figures and Tables

**Table 1:** Composition of faba bean flours used in the study (data from AGT, Canada).

Flour	Moisture	Protein	Starch	Dietary Fiber	Fat
Wheat	≤10%	≤13%	≤70%	≤3%	≤2%
PulsePlus™ faba bean	≤13%	≤29%	≤40%	≤9%	≤4%
PulsePlus™ faba bean V-6000™	≤10%	≤14%	≤55%	≤9%	≤3%
PulsePlus™ faba Bean Protein 60	≤10%	≤60%	≤2%	≤15%	≤4%
PulsePlus™ faba Bean Protein 90	≤10%	≤89%	≤2%	≤2%	≤6.5%

**Table 2:** Composition of flour mixture used in cracker preparation.

Wheat	FB-Flour	FB-Starch	FB-Protein Concentrate	FB-Protein Isolate
100% wheat flour	60% wheat flour + 40% PulsePlus™ faba bean flour	60% wheat flour + 40% PulsePlus™ faba bean flour V-6000™	60% wheat flour + 40% PulsePlus™ faba bean protein 60	60% wheat flour + 40% PulsePlus™ faba bean protein 90

**Table 3:** Concentrations of major constituents of cracker samples.

Sample	Starch (% FW) *	Protein (% FW) *	Fat (% FW) *	Amylose (% FW of Total Starch) *	Total Dietary Fiber (% FW) *
Wheat cracker	61.8 ± 1.0 ^d^	16.2 ± 0.0 ^a^	4.2 ± 0.0 ^a^	38.6 ± 0.9 ^ab^	6.3 ± 0.2 ^a^
FB-flour supplemented cracker	51.6 ± 1.4 ^c^	22.0 ± 0.0 ^b^	7.1 ± 0.0 ^e^	40.2 ± 0.7 ^ab^	8.9 ± 0.1 ^b^
FB-starch supplemented cracker	61.0 ± 0.8 ^d^	16.8 ± 0.0 ^a^	6.1 ± 0.0 ^d^	44.5 ± 3.2 ^b^	6.7 ± 0.4 ^a^
FB-protein concentrate supplemented cracker	38.2 ± 0.8 ^b^	33.8 ± 0.0 ^c^	5.2 ± 0.0 ^c^	36.9 ± 2.8 ^a^	12.1 ± 0.1 ^c^
FB-protein isolate supplemented cracker	33.5 ± 1.0 ^a^	43.0 ± 0.0 ^d^	4.8 ± 0.1 ^b^	38.7 ± 3.2 ^ab^	9.7 ± 0.2 ^b^

* Concentrations are on fresh weight basis (FW) and different letters in the column show significantly different values at *p* ≤ 0.001 (Tuckey’s HSD). All samples have approximately 10% moisture.

**Table 4:** Concentrations of raffinose family oligosaccharides (RFO) in flour samples used as ingredient.

Flour	Total RFO (mmol/100 g FW) *	Raffinose (% FW) *	Stachyose (% FW) *	Verbascose (% FW) *
Wheat	nd	nd	nd	nd
PulsePlus™ faba bean	3.2 ± 0.1 ^c^	1.4 ± 0.0 ^c^	4.2 ± 0.0 ^c^	3.8 ± 0.0 ^c^
PulsePlus™ faba bean V-6000™	2.3 ± 0.1 ^b^	0.8 ± 0.0 ^b^	2.4 ± 0.0 ^b^	3.0 ± 0.0 ^b^
PulsePlus™ faba bean protein 60	4.5 ± 0.0 ^d^	1.9 ± 0.0 ^c^	5.5 ± 0.0 ^d^	3.8 ± 0.0 ^c^
PulsePlus™ faba bean protein 90	1.6 ± 0.3 ^a^	0.2 ± 0.0 ^a^	1.4 ± 0.0 ^a^	2.3 ± 0.1 ^a^

* Concentrations are on fresh weight basis (FW) and different letters in the column show significantly different values at *p* ≤ 0.001. nd: not detected.

**Table 5:** Amylopectin chain-length distribution of starch isolated from wheat and faba bean supplemented cracker samples.

	Amylopectin Fraction			
Sample	A	B1	B2	C
	DP 6–12	DP 13–24	DP 25–36	DP > 37
Wheat cracker	32.7 ± 0.3 ^c^	47.7 ± 0.3 ^a^	11.5 ± 0 ^a^	8.2 ± 0 ^a^
FB-flour supplemented cracker	31.3 ± 0.7 ^b^	49.3 ± 0.9 ^b^	11.2 ± 0.1 ^a^	8.2 ± 0.1 ^a^
FB-starch supplemented cracker	28.1 ± 0.5 ^a^	50.6 ± 0.2 ^c^	12.0 ± 0.3 ^b^	9.2 ± 0.4 ^c^
FB-protein concentrate supplemented cracker	31.8 ± 0.8 ^bc^	48.2 ± 0.3 ^a^	11.4 ± 0.4 ^a^	8.6 ± 0.1 ^b^
FB-protein isolate supplemented cracker	32.3 ± 0.3 ^c^	47.5 ± 0.4 ^a^	11.4 ± 0.1 ^a^	8.9 ± 0.2 ^bc^

Different letters in the column show significantly different values at *p* ≤ 0.001.

**Table 6:** Concentrations of readily digestible- (RDS), slowly digestible- (SDS), and resistant- (RS) starch along with in vitro hydrolytic index (HI) of meal from cracker samples.

Sample	RDS (%) *	RS (%) *	SDS (%) *	*k* (Kinetic Constant)	HI (%)
Wheat cracker	61.4 ± 0.2 ^d^	1.2 ± 0.6 ^a^	37.3 ± 0.6 ^ab^	0.0265 ± 0.0010 ^ab^	100 ^b^
FB-flour supplemented craker	57.1 ± 0.2 ^b^	4.0 ± 0.9 ^bc^	38.8 ± 0.9 ^c^	0.0228 ± 0.0016 ^a^	94.3 ^a^
FB-starch supplemented craker	55.8 ± 0.6 ^a^	6.0 ± 0.5 ^c^	38.2 ± 0.3 ^bc^	0.0222 ± 0.0010 ^a^	91.8 ^a^
FB-protein concentrate supplemented cracker	60.0 ± 0.3 ^c^	3.5 ± 0.2 ^b^	36.5 ± 0.4 ^a^	0.0291 ± 0.0009 ^b^	99.4 ^b^
FB-protein isolate supplemented cracker	59.9 ± 1.0 ^c^	3.2 ± 1.6 ^b^	36.9 ± 0.7 ^ab^	0.0304 ± 0.0043 ^b^	100.1 ^b^

* Concentrations are on fresh weight basis (FW) and different letters in the column show significantly different values at *p* ≤ 0.001 (Tuckey’s HSD).

**Table 7:** Pearson correlation analysis between cracker meal components and meal digestibility parameters (significance level represented as * *p* < 0.05, ** *p* < 0.01, *** *p* < 0.001).

		Digestibility Parameters of Ground Cracker (Meal) Samples				
		HI	*k*	RDS	RS	SDS
Cracker meal components	Total Starch	−0.473 *	−0.681 **	−0.298	0.069	0.511 *
	Protein	0.503 *	0.715 ***	0.327	−0.089	−0.539 *
	Fat	−0.734 ***	−0.623 **	−0.832 ***	0.628 **	0.682 **
	Amylose	−0.604 **	−0.251	−0.452 *	0.484 *	0.125
	TDF	0.34	0.516 *	0.203	0.007	−0.441
	A chain	0.796 ***	0.626 **	0.846 ***	−0.79 ***	−0.434
	B1 chain	−0.895 ***	−0.761 ***	−0.895 ***	0.781 ***	0.554 *
	B2 chain	−0.38	−0.36	−0.407	0.396	0.181
	C chain	−0.251	0.01	−0.425	0.543	−0.033

## Data Availability

Not applicable.
